# Wells syndrome: clinical findings and treatment management in a large cohort of 48 patients

**DOI:** 10.1111/ddg.15930

**Published:** 2026-01-13

**Authors:** Marco Adriano Chessa, Silvia Robuffo, Tullio Brunetti, Luca Rapparini, Bianca Maria Piraccini, Cosimo Misciali, Beatrice Raone, Iria Neri, Federica Filippi

**Affiliations:** ^1^ Dermatology Unit IRCCS Azienda Ospedaliero‐Universitaria di Bologna Bologna Italy; ^2^ Department of Medical and Surgical Sciences Alma Mater Studiorum University of Bologna Bologna Italy

**Keywords:** Eosinophilic cellulitis, eosinophilic granuloma annulare, immunomodulators, inflammatory skin disorder, recurrences in Wells syndrome, Wells syndrome

## Abstract

**Background and Objectives**: Wells syndrome (WS) is a rare inflammatory skin disorder typically characterized by erythematous, edematous, and pruritic plaques. Despite its distinct histopathological features, WS remains an underdiagnosed disease due to its variable clinical presentations and overlap with other dermatological conditions. This study aims to provide a comprehensive overview of WS to enhance diagnostic and management.

**Patients and Methods**: A retrospective analysis for the period 2018‐2023 was conducted on 48 patients with WS at the Dermatology Unit of the University of Bologna, Italy. Data collected included demographics, clinical manifestations, hematological findings, histopathological characteristics, therapies, and outcomes. Descriptive statistics were used to analyze and summarize the data.

**Results**: The mean onset age was 60.7 years, with 56% of patients having localized WS and 44% generalized WS. The most common subtype was urticaria‐like WS (38%), followed by papulonodular, vesiculobullous, granuloma annulare‐like, and cellulitis‐like forms. First‐line treatments included topical and systemic corticosteroids. In contrast, recurrence was observed in 29% of cases during the follow‐up.

**Conclusions**: WS is a heterogeneous underdiagnosed condition requiring clinical‐histologic correlation for accurate diagnosis. Tailored therapeutic strategies are essential, particularly for generalized or refractory cases. Future research should explore the immunological mechanisms underlying WS and identify predictive factors for recurrence.

## INTRODUCTION

Wells syndrome (WS), also referred to as eosinophilic cellulitis, is a rare inflammatory skin disorder thought to result from a type IV hypersensitivity reaction triggered by various exogenous and endogenous factors.^1^, ^2^ Wells syndrome is typically characterized by erythematous, edematous, and pruritic plaques and presents with recurrent lesions on the trunk and extremities. These lesions are non‐tender and may occur with either a localized or generalized distribution.^3^, ^4^ The clinical spectrum of WS encompasses several subtypes, including urticaria‐like, papulonodular, vesiculobullous, granuloma annulare‐like, and cellulitis‐like variants.^3^, ^4^ Despite its distinct histopathological features, WS remains an underdiagnosed condition due to its variable presentations and overlap with other dermatological conditions such as urticaria, cellulitis, and hypersensitivity reactions. The relatively low prevalence of WS contributes further to the challenges in its recognition. In the literature, diagnostic criteria have been proposed to increase diagnostic accuracy (Table 1).^5^

**TABLE 1:** Proposed diagnostic criteria for Wells syndrome.^5^

Major (2 of 4 required)
Clinical variant: Plaque‐type Annular granuloma‐like Urticaria‐like Papulovesicular‐like Bullous Papulonodular Fixed drug eruption‐like
Relapsing‐remittent course
No evidence of systemic disease
Histology: eosinophilic infiltrates, no vasculitis
Minor (at least 1 required)
Flame figures
Histology: Granulomatous change
Peripheral eosinophilia not persistent and not greater than >1500/uL
Triggering factor

The objective of this study is to provide a comprehensive overview of the epidemiological, clinical, histopathological, and therapeutic characteristics of WS, while offering insights into potential areas for further research.

## MATERIALS AND METHODS

This retrospective study was conducted at the Dermatology Unit of the University of Bologna, Italy, according to the Helsinki Declaration and patients signed written informed consent to participate. The study was approved by local Ethical Committee (protocol n°607/2024 Oss/AOUBo). Patients with a diagnosis of WS between January 2018 to December 2023 were included. Inclusion criteria encompassed a confirmed diagnosis based on a combination of clinical presentation and histopathological findings and a follow‐up period of at least 6 months. Data regarding demographics, clinical manifestations, laboratory findings, histopathological features, therapeutic interventions, and outcomes were recorded.

The data collection process was thorough and included detailed documentation of clinical photographs, histopathological slides, and treatment regimens. Statistical methods were applied to summarize the data, with continuous variables expressed as means ± standard deviations and categorical variables presented as percentages.

## RESULTS

**FIGURE 1:**
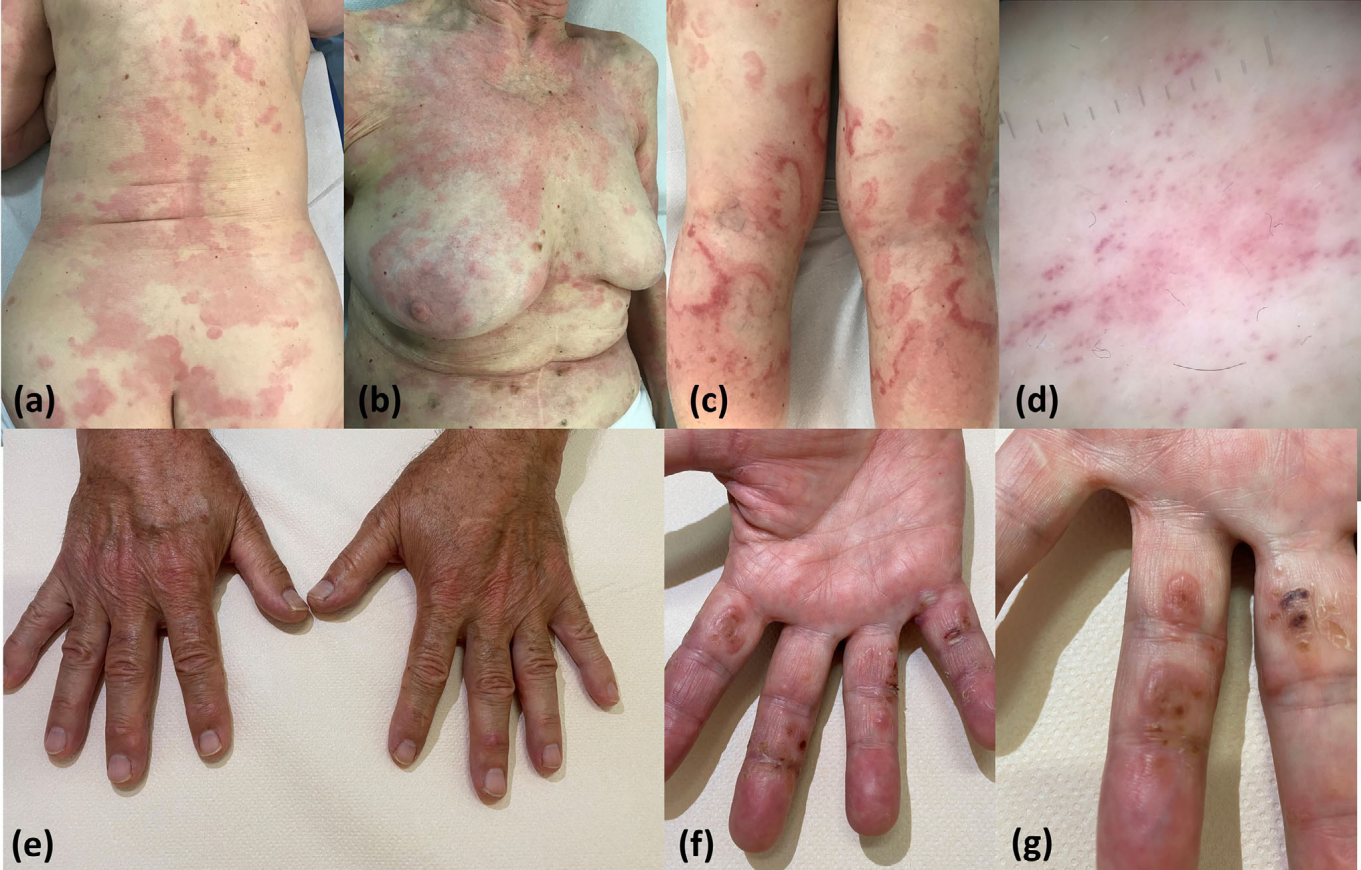
Urticaria‐like variant of Wells syndrome with erythematous figurate edematous borders. (a) Back, (b) thorax, (c) lower limbs. (d) Dermoscopy (× 10) showing purpuric dots on a reddish background. Granuloma annulare‐like variant of Wells syndrome. (e) Erythematous‐orange papules, nodules, and plaques on the back of the hands. (f, g) Palms.

Our cohort consisted of 48 patients, with a mean age of onset of 60.7 ± 20.1 years (Table 2). The male‐to‐female ratio was nearly equal, with 25 males (52%) and 23 females (48%). WS presented in two major forms: generalized and localized. Generalized WS was reported in 21 patients (44%) (Figure 1a–c). Localized WS was observed in 27 patients (56%), predominantly affecting acral sites such as the upper and lower limbs in 15 cases (56%). The head and trunk were involved in nine cases (33%), while the trunk alone was affected in three cases (11%).

**TABLE 2:** Epidemiological, clinical, and management features of Wells syndrome in 48 patients.

Characteristics of patients	n (%)
***Epidemiological Findings***	
Age at onset, mean ± SD	60.7 ± 20.1
Male gender	25 (52%)
***Symptoms***	
Itch	46 (96%)
Systemic symptoms (fever, malaise, arthralgia)	8 (16%)
***Clinical severity***	
Localized form	27 (56%)
Generalized form	21 (44%)
***Clinical type***	
Urticaria‐like	18 (38%)
Papulonodular	12 (25%)
Vesiculo‐bullous	10 (21%)
Granuloma annulare‐like	5 (10%)
Cellulitis like/plaque	3 (6%)
***Hematological abnormalities***	
Eosinophilia (> 0.5 × 10^9^/l)	29 (60%)
Leukocytosis (> 11 × 10^9^/l)	15 (31%)
***Histopathological findings***	
Eosinophilic infiltration in the superficial and deep dermis	48 (100%)
Flame figures	34 (71%)
***Trigger factors***	
Trauma	4 (8%)
Infection	2 (4%)
Insect bites	4 (8%)
Vaccines	2 (4%)
Drugs	1 (2%)
Unknown	35 (73%)
***Therapy***	
Oral Antihistamines	48 (100%)
Topical corticosteroids	48 (100%)
Systemic corticosteroids	15 (31%)
Oral immunomodulators	8 (17%)
Methotrexate	2 (4%)
PUVA therapy	3 (6%)
NB‐UVB therapy	1 (2%)
***Clinical course***	
Complete remission	34 (71%)
Recurrence	14 (29%)

**FIGURE 2:**
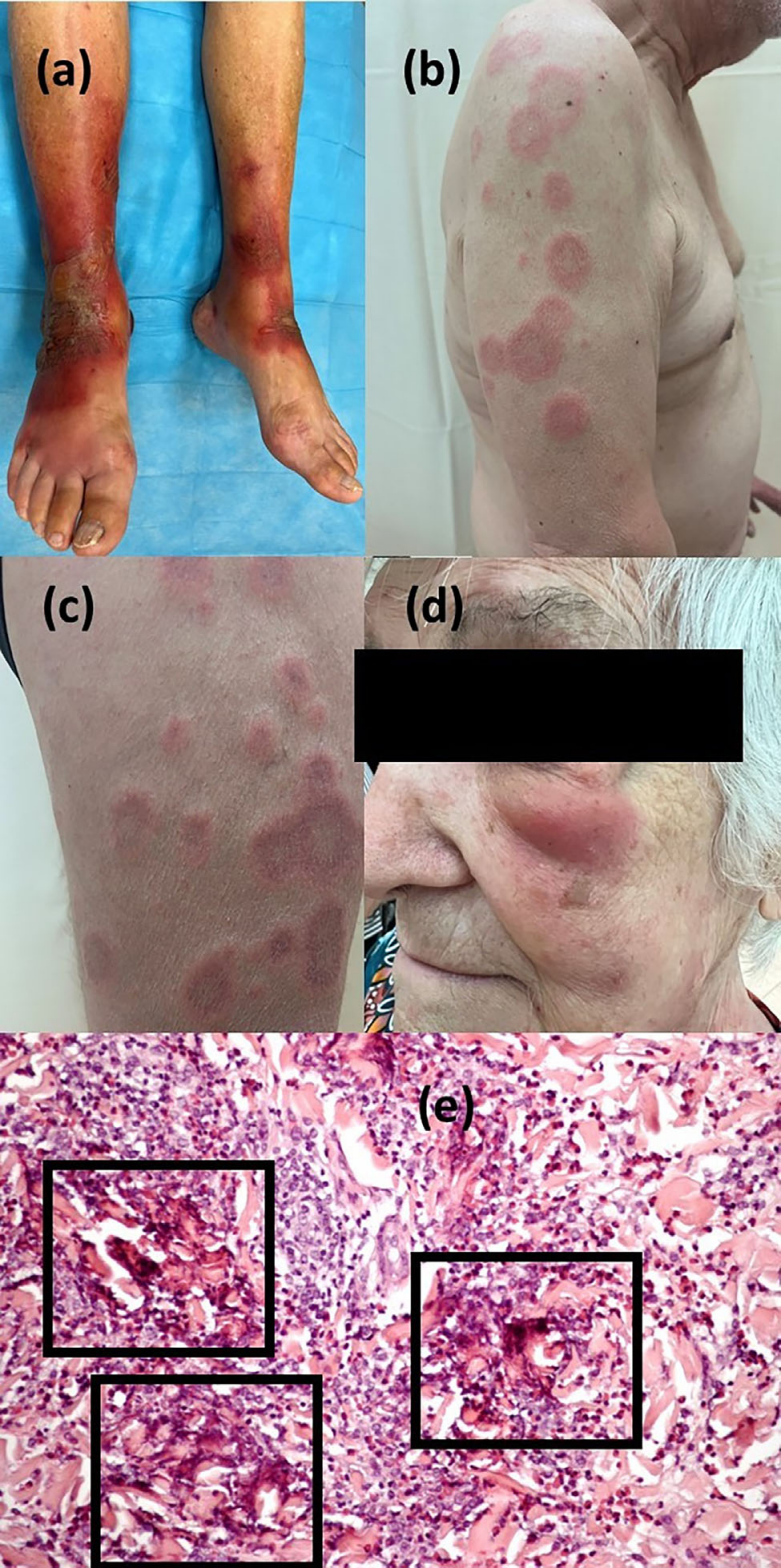
Bullous erysipelas‐like variant of Wells syndrome. (a) Painful and erythematous bullous lesions with a sharp border on the lower limbs. Granuloma annulare‐like variant of Wells syndrome. (b, c) Diffuse lesions of the trunk and upper limbs. Erysipelas‐like variant of Wells syndrome. (d) Painful edematous lesion on the lower left eyelid. (e) Histologic findings showing a dermal infiltrate composed of eosinophils and histiocytes around amorphous collagen depositions, forming the typical flame‐shaped area within the black box (hematoxylin‐eosin staining, original magnification × 300).

The clinical manifestations of WS were heterogeneous, with the urticaria‐like subtype being the most prevalent, accounting for 18 cases (38%). This form was characterized by edematous borders and purpuric dots, which were evident on dermoscopy and attributed to extravasated erythrocytes (Figure 1a–d). Other subtypes included papulonodular (12 cases, 25%; Figure 1e–g), vesiculobullous (10 cases, 21%; Figure 2a), granuloma annulare‐like (5 cases, 10%; Figure 2b, c), and cellulitis‐like (3 cases, 6%; Figure 2d). Pruritus was a common symptom, affecting 96% of the patients, while systemic symptoms such as fever, malaise, and arthralgia were reported in 16% of cases.

Hematological abnormalities were identified in our cohort. Peripheral eosinophilia, defined as an eosinophil count ≥ 0.5 × 10^9^/l, was present in 29 patients (60%), and leukocytosis (≥ 11 × 10^9^/l) was observed in 15 patients (31%). Histopathological examination revealed eosinophilic infiltration in the superficial and deep dermis in all cases, along with the characteristic “flame figures” in 34 patients (71%) (Figure 2e). Granulomatous infiltration was less common, observed in 10% of cases, but its presence underscores the histopathological diversity of WS and its overlap with other granulomatous disorders.

Potential trigger factors were reported in 13 patients (27%). Trauma and insect bites were the most frequently reported triggers, each accounting for 8% of cases, followed by infections (4%), vaccines (4%), and drugs (2%). However, in most cases (73%), no specific trigger was identified, reflecting the idiopathic nature of Wells syndrome in many instances.

Therapeutic management varied based on the severity and extent of WS. All patients received oral antihistamines and topical corticosteroids as first‐line treatments. Systemic corticosteroids were administered to 15 patients (31%), with methylprednisolone (0.3–0.5 mg/kg bodyweight [BW]/day) as the starting dose, followed by gradual tapering over 2–3 weeks. Clinical remission, defined as the absence of new lesions for at least 6 months, was achieved in 34 patients (71%). Recurrences occurred in 14 patients (29%) during a mean follow‐up period of 12 ± 6 months. The 14 recurrences, according to the clinical forms of Wells syndrome, were six of 18 (33%) urticaria‐like, three of five (60%) granuloma annulare‐like, three of ten (30%) papulonodular, and two of ten (20%) vesiculobullous. In recalcitrant WS, additional therapies such as phototherapy in four cases (8%) and corticosteroid‐sparing agents in ten cases (21%) were prescribed. These agents included hydroxychloroquine in 5 patients, methotrexate in two patients, and dapsone in three patients. Transitory sclerodermiform‐like pigmented areas were reported in six patients (13%), resolving within 6–12 weeks.

Epidemiological, clinico‐pathological and therapeutical features of each patient are better specified in Table 3.

**TABLE 3:** Epidemiological, clinicopathological, and therapeutic features of each affected patient (n = 48).

Sex	Year of diagnosis	Age of onset	Clinical presentation	Clinical extent	Biopsy site	Trigger factors	Hematological abnormalities	Histopathological findings	Symptoms	Treatment	Clinical course
F	2018	84	Vesiculobullous	Localized	Lower limbs	Unknown	None	Eosinophilic infiltration in the superficial and deep dermis and characteristic "flame figures"	Itching	Topical corticosteroids and antihistamines	Remission
F	2023	75	Urticaria‐like	Generalized	Lower limbs	Trauma	Eosinophilia	Eosinophilic infiltration in the superficial and deep dermis	Itching	Systemic corticosteroids TRopical corticosteroids + antihistamines + NB‐UVB therapy	Recurrent
F	2023	61	Urticaria‐like	Generalized	Abdomen	Infection	Leukocytosis	Eosinophilic infiltration in the superficial and deep dermis	Itching + systemic symptoms	Systemic corticosteroids + antihistamine + topical corticosteroids + antihistamines + methotrexate	Recurrent
M	2022	80	Plaque/cellulitis like	Generalized	Back	Insect bite	Eosinophilia	Eosinophilic infiltration in the superficial and deep dermis	Asymptomatic	Topical corticosteroids + antihistamines	Remission
F	2020	68	Urticaria‐like	Generalized	Abdomen	Vaccine	Leukocytosis	Eosinophilic infiltration in the superficial and deep dermis and characteristic “flame figures”	Itching	Systemic corticosteroids + Topical corticosteroids + antihistamines + oral immunomodulators (dapsone)	Recurrent
F	2019	55	Granuloma‐annulare like	Generalized	Abdomen	Drug	Eosinophilia	Eosinophilic infiltration in the superficial and deep dermis and characteristic “flame figures”	Itching	Topical corticosteroids + antihistamines	Remission
M	2019	64	Papulonodular	Generalized	Lower limbs	Trauma	Eosinophilia	Eosinophilic infiltration in the superficial and deep dermis and characteristic “flame figures”	Itching	Systemic corticosteroids + Topical corticosteroids + antihistamines + methotrexate	Recurrent
M	2019	91	Urticaria‐like	Generalized	Back	Vaccine	Eosinophilia	Eosinophilic infiltration in the superficial and deep dermis and characteristic “flame figures”	Itching + systemic symptoms	Systemic corticosteroids + topical corticosteroids + antihistamines + oral immunomodulators (hydroxychloroquine)	Recurrent
M	2019	81	Vesiculobullous	Generalized	Lower limbs	Unknown	Leukocytosis	Eosinophilic infiltration in the superficial and deep dermis and characteristic “flame figures”	Itching	Systemic corticosteroids + topical corticosteroids + antihistamines + oral immunomodulators (dapsone)	Recurrent
M	2018	74	Papulonodular	Localized	Back	Insect bite	Eosinophilia	Eosinophilic infiltration in the superficial and deep dermis and characteristic “flame figures”	Itching	Topical corticosteroids + antihistamines	Remission
M	2021	41	Urticaria‐like	Generalized	Abdomen	Trauma	Eosinophilia	Eosinophilic infiltration in the superficial and deep dermis and characteristic “flame figures”	Itching	Topical corticosteroids + antihistamines	Remission
M	2021	75	Papulonodular	Generalized	Abdomen	Unknown	Leukocytosis	Eosinophilic infiltration in the superficial and deep dermis and characteristic “flame figures”	Itching + systemic symptoms	Systemic corticosteroids + topical corticosteroids + antihistamines + oral immunomodulators (dapsone)	Recurrent
M	2020	39	Papulonodular	Generalized	Upper limbs	Unknown	Eosinophilia	Eosinophilic infiltration in the superficial and deep dermis and characteristic “flame figures”	Itching	Topical corticosteroids + antihistamines	Remission
M	2020	85	Urticaria‐like	Generalized	Abdomen	Unknown	Eosinophilia	Eosinophilic infiltration in the superficial and deep dermis and characteristic “flame figures”	Itching + systemic symptoms	Systemic corticosteroids + topical corticosteroids + antihistamines + oral immunomodulators (hydroxychloroquine)	Recurrent
F	2019	19	Vesiculobullous	Generalized	Lower limbs	Unknown	Eosinophilia	Eosinophilic infiltration in the superficial and deep dermis and characteristic “flame figures”	Itching	Topical corticosteroids + antihistamines	Remission
F	2018	76	Granuloma annulare‐like	Generalized	Lower limbs	Trauma	None	Eosinophilic infiltration in the superficial and deep dermis	Itching	Systemic corticosteroids + topical corticosteroids + antihistamines oral immunomodulators (hydroxychloroquine)	Recurrent
F	2019	14	Vesiculobullous	Localized	Lower limbs	Unknown	Eosinophilia	Eosinophilic infiltration in the superficial and deep dermis and characteristic “flame figures”	Itching + systemic symptoms	Systemic corticosteroids + topical corticosteroids + antihistamines	Remission
F	2018	41	Vesiculobullous	Localized	Chest	Unknown	Eosinophilia	Eosinophilic infiltration in the superficial and deep dermis and characteristic “flame figures”	Itching	Topical corticosteroids + antihistamines	Remission
F	2021	76	Urticaria‐like	Generalized	Abdomen	Unknown	Eosinophilia	Eosinophilic infiltration in the superficial and deep dermis and characteristic “flame figures”	Itching + Systemic symptoms	Systemic corticosteroids + topical corticosteroids + antihistamines + oral immunomodulators (hydroxychloroquine)	Recurrent
F	2023	89	Urticaria‐like	Generalized	Lower limbs	Unknown	None	Eosinophilic infiltration in the superficial and deep dermis and characteristic “flame figures”	Itching	Topical corticosteroids + antihistamines	Remission
M	2023	74	Granuloma annulare‐like	Generalized	Hand	Unknown	Eosinophilia	Eosinophilic infiltration in the superficial and deep dermis and characteristic “flame figures”	Itching	Systemic corticosteroids + topical corticosteroids + antihistamines PUVA	Recurrent
M	2022	62	Plaque/cellulitis like	Generalized	Abdomen	Insect bite	Eosinophilia	Eosinophilic infiltration in the superficial and deep dermis and characteristic “flame figures”	Itching	Topical corticosteroids + antihistamines	Remission
F	2020	102	Urticaria‐like	Generalized	Back	Unknown	None	Eosinophilic infiltration in the superficial and deep dermis and characteristic “flame figures”	Itching	Topical corticosteroids + antihistamines	Remission
F	2023	60	Urticaria‐like	Localized	Upper limbs	Unknown	Eosinophilia Leukocytosis	Eosinophilic infiltration in the superficial and deep dermis	Itching	Topical corticosteroids + antihistamines	Remission
M	2023	73	Papulonodular	Localized	Abdomen	Unknown	Eosinophilia	Eosinophilic infiltration in the superficial and deep dermis and characteristic “flame figures”	Itching	Topical corticosteroids + antihistamines	Remission
F	2022	28	Vesiculobullous	Localized	Abdomen	Unknown	Eosinophilia	Eosinophilic infiltration in the superficial and deep dermis and characteristic “flame figures”	Itching	Topical corticosteroids + antihistamines	Remission
F	2022	81	Vesiculobullous	Localized	Abdomen	Unknown	Eosinophilia	Eosinophilic infiltration in the superficial and deep dermis and characteristic “flame figures”	Itching + systemic symptoms	Topical corticosteroids + antihistamines	Remission
F	2022	62	Urticaria‐like	Localized	Back	Unknown	Eosinophilia	Eosinophilic infiltration in the superficial and deep dermis and characteristic “flame figures”	Itching	Topical corticosteroids + antihistamines	Remission
M	2022	41	Plaque/cellulitis like	Localized	Abdomen	Unknown	None	Eosinophilic infiltration in the superficial and deep dermis and characteristic “flame figures”	Asymptomatic	Topical corticosteroids + antihistamines	Remission
M	2022	70	Papulonodular	Localized	Lower limbs	Infection	Leukocytosis	Eosinophilic infiltration in the superficial and deep dermis	Itching	Topical corticosteroids + antihistamines	Remission
F	2021	58	Granuloma annulare‐like	Localized	Lower limbs	Insect bite	Eosinophilia	Eosinophilic infiltration in the superficial and deep dermis and characteristic “flame figures”	Itching	Topical corticosteroids + antihistamines	Remission
F	2021	70	Urticaria‐like	Localized	Back	Unknown	Eosinophilia	Eosinophilic infiltration in the superficial and deep dermis and characteristic “flame figures”	Itching	Topical corticosteroids + antihistamines	Remission
F	2021	50	Papulonodular	Localized	Upper limbs	Unknown	Leukocytosis	Eosinophilic infiltration in the superficial and deep dermis and characteristic “flame figures”	Itching	Topical corticosteroids + antihistamines	Remission
M	2021	34	Papulonodular	Localized	Abdomen	Unknown	Eosinophilia	Eosinophilic infiltration in the superficial and deep dermis and characteristic “flame figures”	Itching	Topical corticosteroids + antihistamines	Remission
M	2021	64	Urticaria‐like	Localized	Abdomen	Unknown	None	Eosinophilic infiltration in the superficial and deep dermis	Itching	Topical corticosteroids + antihistamines	Remission
M	2021	40	Urticaria‐like	Localized	Lower limbs	Unknown	Eosinophilia Leukocytosis	Eosinophilic infiltration in the superficial and deep dermis and characteristic “flame figures”	Itching	Topical corticosteroids + antihistamines	Remission
F	2020	52	Urticaria‐like	Localized	Abdomen	Unknown	Leukocytosis	Eosinophilic infiltration in the superficial and deep dermis	Itching	Topical corticosteroids + antihistamines	Remission
M	2020	41	Vesiculobullous	Localized	Buttock	Unknown	None	Eosinophilic infiltration in the superficial and deep dermis and characteristic “flame figures”	Itching	Topical corticosteroids + antihistamines	Remission
M	2020	83	Urticaria‐like	Localized	Back	Unknown	None		Itching	Topical corticosteroids + antihistamines	Remission
M	2020	37	Urticaria‐like	Localized	Hand	Unknown	Eosinophilia Leukocytosis	Eosinophilic infiltration in the superficial and deep dermis and characteristic “flame figures”	Itching	Topical corticosteroids + antihistamines	Remission
M	2020	22	Urticaria‐like	Localized	Back	Unknown	None	Eosinophilic infiltration in the superficial and deep dermis and characteristic “flame figures”	Itching	Topical corticosteroids + antihistamines	Remission
M	2019	65	Vesiculobullous	Localized	Lower limbs	Unknown	Eosinophilia Leukocytosis	Eosinophilic infiltration in the superficial and deep dermis	Itching	Topical corticosteroids + antihistamines	Remission
M	2019	53	Papulonodular	Localized	Neck	Unknown	Eosinophilia	Eosinophilic infiltration in the superficial and deep dermis	Itching	Topical corticosteroids + antihistamines	Remission
M	2019	87	Papulonodular	Localized	Lower limbs	Unknown	Leukocytosis	Eosinophilic infiltration in the superficial and deep dermis and characteristic “flame figures”	Itching	Topical corticosteroids + antihistamines	Remission
M	2018	60	Papulonodular	Localized	Lower limbs	Unknown	Eosinophilia Leukocytosis	Eosinophilic infiltration in the superficial and deep dermis	Itching + systemic symptoms	Systemic corticosteroids Topical corticosteroids + antihistamines + oral immunomodulators + oral immunomodulators (hydroxychloroquine)	Recurrent
F	2020	50	Granuloma annulare‐like	Generalized	Abdomen	Unknown	Leukocytosis	Eosinophilic infiltration in the superficial and deep dermis	Itching	Systemic corticosteroids Topical corticosteroids + antihistamines + PUVA	Recurrent
F	2019	49	Vesiculobullous	Generalized	Chest	Unknown	Eosinophilia Leukocytosis	Eosinophilic infiltration in the superficial and deep dermis and characteristic “flame figures”	Itching	Systemic corticosteroids Topical corticosteroids + antihistamines + PUVA	Recurrent
F	2018	61	Papulonodular	Localized	Abdomen	Unknown	None	Eosinophilic infiltration in the superficial and deep dermis	Itching	Topical corticosteroids + antihistamines	Remission

## DISCUSSION

The concept of WS as a distinct entity has been repeatedly questioned over time, due to its high clinical variability and suggestive, but not pathognomonic histopathological features. Some reports of WS in association with rare multisystem eosinophilic disorders, such as Churg‐Strauss syndrome and hypereosinophilic syndrome, further brought into question the existence of WS as a distinct disease, though a possible explanation could be the central role played by the overactivation of eosinophils in all these conditions.^5^

We support the concept of Wells syndrome as a distinct entity, which accounts for common characteristics in affected patients, such as the exclusively cutaneous involvement and the benign course.

The findings of this study provide valuable insights into the clinical and histopathological characteristics of WS, as well as its therapeutic outcomes. The predominance of the urticaria‐like subtype with purpuric dots at dermoscopy highlights its diagnostic significance and potential role in differentiating WS from other dermatological conditions such as urticaria and urticarial vasculitis.^6^, ^7^ Furthermore, cases of chronic spontaneous urticaria concomitant with WS have also been reported.^6^

The observed variability in clinical presentations, ranging from localized to generalized forms and encompassing rare subtypes like granuloma annulare‐like and cellulitis‐like variants, underscores the diagnostic challenges posed by WS.^4^, ^5^, ^6^, ^7^, ^8^, ^9^, ^10^, ^11^ Furthermore, the annular granuloma‐like form has been reported in the literature both as a clinical variant of WS and also as a distinctive rare condition known as eosinophilic granuloma annulare.^13^, ^14^ El‐Khalawany M. et al. reported eosinophilic granuloma annulare as a rare form of WS characterized by a chronic course, resistance to treatment and high relapse rate.^14^ In our case series, the odds ratio for patients with granuloma annulare‐like WS was approximately two times higher compared to the other clinical forms.

Histopathological examination remains a cornerstone in the diagnosis of WS. The presence of eosinophilic granulomatous infiltration and flame figures, while characteristic, is not pathognomonic, necessitating clinical‐histologic correlation for accurate diagnosis. Differential diagnoses, including insect bites, bullous pemphigoid, and drug hypersensitivity reactions, should always be considered.^1^, ^2^, ^3^ The clinical features seem to depend on the location of the dermal infiltrate and also on the timing of the skin biopsy.^1^, ^3^, ^15^

The therapeutic approach to WS is equally nuanced. The treatment is tailored to the severity and progression of the disease, with a focus on managing inflammation and preventing flare‐ups. The main therapeutic approaches for Wells syndrome include topical or systemic steroids to reduce inflammation and control symptoms; oral antihistamines to reduce itching; antibiotics if secondary bacterial infection is suspected due to the skin lesions; immunosuppressive agents in cases where corticosteroids are not effective or when the condition relapses. While topical corticosteroids suffice for localized cases, systemic corticosteroids are often required for generalized or refractory forms.^2^, ^4^, ^12^ The recurrence rate of 29% observed in our study aligns with previous reports, highlighting the chronic‐relapsing nature of WS over years.^2^ In steroid‐resistant cases or if steroids are not tolerated by the patient, cyclosporine, dapsone, azathioprine, griseofulvin, doxycycline, minocycline, hydroxychloroquine, methotrexate, topical tacrolimus, and PUVA therapy have been reported as effective.^13^ In addition, targeted therapies such as anti–TNF‐α agents, omalizumab, and mepolizumab have also been reported as useful.^7^, ^16^, ^17^, ^18^, ^19^

Our case series is larger than those previously reported in the literature, and several explanations can be considered: *(1)* our unit is a referral center for immune‐mediated diseases, which allows us to receive patients from a wide geographical area; *(2)* we have the opportunity to perform on‐site skin biopsies, which are analyzed in a dermatopathology laboratory and are particularly helpful in conditions with self‐limiting or recurrent courses, such as Wells syndrome; *(3)* a dermatological emergency service with direct patient access is available, enabling the clinical presentation to be evaluated during the acute phase.

## Conclusions

Wells syndrome is a rare but likely underdiagnosed condition due to the variety of clinical presentations. Histopathological examination and clinical‐histologic correlation are indispensable for diagnosis, while therapeutic strategies should be tailored to individual patient needs. Despite advancements in understanding WS, relapses remain a significant concern, longitudinal studies with larger cohorts are needed to better understand the natural history of WS and identify predictive factors for recurrence.

## CONFLICT OF INTEREST STATEMENT

None.

## References

[1] Moossavi M , Mehregan DR . Wells’ syndrome: a clinical and histopathologic review of seven cases. Int J Dermatol. 2003;42(1):62‐67. 12581147 10.1046/j.1365-4362.2003.01705.x

[2] Sinno H , Lacroix JP , Lee J , et al. Diagnosis and management of eosinophilic cellulitis (Wells’ syndrome): A case series and literature review. Can J Plast Surg. 2012;20(2):91‐97. 23730155 10.1177/229255031202000204 PMC3383552

[3] Consigny S , Courville P , Young P , et al. [Histological and clinical forms of the eosinophilic cellulitis]. Ann Dermatol Venereol. 2001;128(3 Pt 1):213‐216. 11319382

[4] Caputo R , Marzano AV , Vezzoli P , Lunardon L . Wells syndrome in adults and children: a report of 19 cases. Arch Dermatol. 2006;142(9):1157‐1161. 16983003 10.1001/archderm.142.9.1157

[5] Heelan K , Ryan JF , Shear NH , Egan CA . Wells syndrome (eosinophilic cellulitis): Proposed diagnostic criteria and a literature review of the drug‐induced variant. J Dermatol Case Rep. 2013;7(4):113‐120. 24421864 10.3315/jdcr.2013.1157 PMC3888780

[6] Marzano AV , Maronese CA , Genovese G , et al. Urticarial vasculitis: Clinical and laboratory findings with a particular emphasis on differential diagnosis. J Allergy Clin Immunol. 2022;149(4):1137‐1149. 35396080 10.1016/j.jaci.2022.02.007

[7] Ogueta I , Spertino J , Deza G , et al. Wells syndrome and chronic spontaneous urticaria: report of four cases successfully treated with omalizumab. J Eur Acad Dermatol Venereol. 2019;33(10):e388‐e391. 31106467 10.1111/jdv.15683

[8] Gallard C , Law‐Ping‐Man S , Darrieux L , et al. [Wells syndrome mimicking facial cellulitis: Three cases]. Ann Dermatol Venereol. 2017;144(4):284‐289. 27839729 10.1016/j.annder.2016.09.676

[9] Deniz M , Demir‐Önder K , Özkaraman Y , et al. Clinical Entity Mimicking Infectious Cellulitis: Eosinophilic Cellulitis (Wells’ Syndrome). Infect Dis Clin Microbiol. 2023;5(4):376‐379. 38633856 10.36519/idcm.2023.279 PMC10986720

[10] Ghislain PD , Van Eeckhout P . Eosinophilic cellulitis of papulonodular presentation (Wells’ syndrome). J Eur Acad Dermatol Venereol. 2005;19(2):226‐227. 15752298 10.1111/j.1468-3083.2005.01027.x

[11] Katoulis AC , Bozi E , Samara M , et al. Idiopathic bullous eosinophilic cellulitis (Wells’ syndrome). Clin Exp Dermatol. 2009;34(7):e375‐e376. 19489853 10.1111/j.1365-2230.2009.03328.x

[12] Guglielmo A , Filippi F , Pileri A , et al. Bullous Wells Syndrome: a needle in the haystack. Int J Dermatol. 2021;60(4):e150‐e153. 33259051 10.1111/ijd.15250

[13] Żychowska M , Tutka K , Reich A . Mepolizumab Therapy for Recalcitrant Eosinophilic Annular Erythema in an Adult: A Case Report and Review of Treatment Options. Dermatol Ther (Heidelb). 2020;10(4):893‐899. 32578132 10.1007/s13555-020-00412-9 PMC7308446

[14] El‐Khalawany M , Al‐Mutairi N , Sultan M , Shaaban D . Eosinophilic annular erythema is a peculiar subtype in the spectrum of Wells syndrome: a multicentre long‐term follow‐up study. J Eur Acad Dermatol Venereol. 2013;27(8):973‐979. 22731886 10.1111/j.1468-3083.2012.04616.x

[15] Weiss G , Shemer A , Confino Y , et al. Wells’ syndrome: report of a case and review of the literature. Int J Dermatol. 2001;40(2):148‐152. 11328401

[16] Terhorst‐Molawi D , Altrichter S , Röwert J , et al. Effective treatment with mepolizumab in a patient with refractory Wells syndrome. J Dtsch Dermatol Ges. 2020;18(7):737‐739. 10.1111/ddg.14151 32713149

[17] Räßler F , Lukács J , Elsner P . Treatment of eosinophilic cellulitis (Wells syndrome) – a systematic review. J Eur Acad Dermatol Venereol. 2016;30(9):1465‐1479. 27357601 10.1111/jdv.13706

[18] Trica OC , Mocan LP , Boit‐Trapcea R . Mepolizumab is highly effective in a rare case of Wells syndrome: Eosinophilic granulomatosis with polyangiitis overlap. Dermatol Ther. 2022;35(11):e15799. 36045257 10.1111/dth.15799

[19] Herout S , Bauer WM , Schuster C , Stingl G . Eosinophilic cellulitis (Wells syndrome) successfully treated with mepolizumab. JAAD Case Rep. 2018;4(6):548‐550. 29892672 10.1016/j.jdcr.2018.02.011 PMC5991892

